# Editorial: Zoonotic Microorganisms and Spread of Acquired Polymyxin Resistance Determinants

**DOI:** 10.3389/fmicb.2022.849316

**Published:** 2022-02-04

**Authors:** Azucena Mora, Maria Jorge Campos, Alberto Quesada

**Affiliations:** ^1^Laboratorio de Referencia de Escherichia coli, Dpto. de Microbioloxía e Parasitoloxía, Universidade de Santiago de Compostela, Lugo, Spain; ^2^Marine and Environmental Sciences Centre, School of Tourism and Maritime Technology (ESTM), Polytechnic of Leiria, Peniche, Portugal; ^3^Departamento de Bioquímica, Facultad de Veterinaria, Universidad de Extremadura, Cáceres, Spain

**Keywords:** colistin resistance, enterobacteria, zoonotic agent, antimicrobial resistance (AMR), MCR genes

Currently, the use of colistin as a last-resort antibiotic in human medicine is highly compromised due to the continuous emergence of resistant enterobacteria with acquired determinants like plasmid-mediated resistance genes (*mcr*), mutations that activate the PmrAB system, or still unknown mechanisms. On the other hand, the correlation between the individual, or combined activity of these mechanisms, with the functional expression of resistance to colistin is still poorly understood. Based on a One-Health approach, this topic aimed to gather knowledge in the transmission and expression of colistin resistance determinants in Gram-negative bacteria isolated from different sources.

Since the *mcr-1* plasmid-mediated colistin resistance gene was discovered in China in 2015 (Liu et al., 2016), numerous surveys have been performed worldwide to monitor the prevalence and spread of *mcr* in food animals. Industrial farming, particularly poultry and swine, have been highlighted as a zoonotic source of *mcr* (mainly *mcr-1*) *Escherichia coli* isolates (Irrgang et al., 2016; García-Meniño et al., 2018). Cheng et al. analyzed 200 fecal swabs collected from six swine farms in northeastern China between July 2016 and June 2017. A total of 176 *E. coli* strains were isolated and the prevalence of *mcr-1* in colistin-resistant *E. coli* was 53.33% (56/105). *mcr-1-*positive *E. coli* showed extensive antimicrobial resistance (AMR) profiles with the presence of additional resistance genes, increased expression of multidrug efflux pump-associated genes, and increased biofilm formation ability. The results of growth assay, competition experiment, and plasmid stability testing showed that acquisition of *mcr-1-*harboring plasmids could reduce the fitness of bacterial hosts, but *mcr-1* remained stable in the recipient strain.

The availability of accurate systems for the diagnosis of AMR is a cornerstone both for its clinical study and for the investigation of the propagation of clones with low susceptibility to colistin. Zhu et al. analyzed the performance of three mainstream commercial antimicrobial susceptibility testing (VITEK 2® COMPACT, Phoenix™ M50, and Bio-kont) in parallel to standard broth microdilution, finding out slight but significant differences among them, especially for enterobacteria like *Escherichia* spp., *Klebsiella* spp., and *Citrobacter* spp. Retrospective studies on frozen stored collections of samples are being used to investigate the evolution of new threats, like plasmid-mediated colistin resistance determinants. Miguela-Villoldo et al. showed that an SYBR Green qPCR assay designed to detect *mcr-1* in pig caecum samples is the best option to provide a highly representative frame of the initial population present in the sample, and although the freeze-thaw process affects bacterial viability, culture-based methods might be a useful complement to study colistin resistance levels.

Poultry and poultry meat have also been highlighted as important contributors to the global antimicrobial burden with potential transmission risk for consumers (Díaz-Jiménez et al., 2021). In Lebanon, where the first *mcr-1*-positive *E. coli* found in poultry dates to 2015, Hiba Al-Mir et al. report a prevalence of 84.4% *mcr-1* positive poultry farms across three Lebanese governorates. The study was conducted on poultry samples collected in 2018 in one large slaughterhouse and originated from 32 individual farms. Furthermore, numerous associated resistances were identified, including the presence of *bla*_*CTX*−*M*_ or *bla*_*CMY*_ genes, and the *mcr-1* gene was mostly located in IncX4 (*n* = 36) and IncI2 (*n* = 24) plasmids.

The findings of Nakano et al. in Japan uncover the potential circulation of *mcr-1* mediated colistin-resistant *E. coli* among livestock and farmers. The authors analyzed fecal samples from 295 healthy livestock (202 cattle and 93 swine) and 62 healthy livestock farmers (53 cattle farmers and nine swine farmers) collected between 2013 and 2015, from 72 livestock farms. The prevalence of *mcr-1*-harboring *E. coli* was 25 (8.47%) of the 295 livestock from 11 farms, and 3.77% (2/53 strains) for cattle farmers, and 11.11% (1/9 strains) for swine farmers. Considering the isolates obtained from livestock and farmers, in four farms, nine isolates had the same genotypical characteristics (sequence types and pulsed-field gel electrophoresis band patterns), plasmid characteristics (incompatibility group and plasmid transferability), and minimum inhibitory concentrations.

Due to the high number of colonized animals, slaughterhouses might represent a significant source of the introduction of *mcr* genes into the food chain through possible contamination of carcasses and products. Furthermore, these bacteria might accumulate in process waters and wastewater from slaughterhouses, contributing to a broad spread of the resistance to other environmental ecosystems including surface waters. The study of Savin et al. supports the necessity of implementing advanced wastewater treatment technologies to limit the contamination of the environment with bacteria expressing resistances against last resort antimicrobials. The authors performed a study to evaluate the occurrence of colistin-resistant *Enterobacteriaceae* in process waters and wastewater from two poultry and two pig slaughterhouses in Germany. Their findings demonstrated a high occurrence of colistin-resistant *E. coli* and *Klebsiella pneumoniae* carrying *mcr-1* on transferable plasmids (incompatibility groups IncI1, IncHI2, IncX4, IncF, and IncI2) in poultry and pig slaughterhouses and indicate their dissemination into surface water. Environmental contamination with colistin-resistant enterobacteria carrying *mcr* genes is also described by Lopes et al., who detected the ST131 clone of *E. coli* isolated from a kale crop in Brazil. In addition to *mcr-1, bla*_*CTX*−*M*−15_, and *qnrB19* genes were associated with IncH12, IncF, and ColE1 replicons, respectively, the two first harboring additional AMR determinants and being efficiently transferred by conjugation.

Wild birds play a key role in the spread of AMR due to their environmental exposure through ingested food or polluted water. In this respect, the results of Zhang et al. provide evidence that migratory birds are potential transmitters of AMR. The authors identified 22 *mcr-1*-positive *E. coli* from 303 *Anser indicus* fecal samples (7.3%) collected in the Guangdong province of China. Coexisting with 24 other types of AMR genes, *mcr-1* was located on IncX4, IncI2, and IncP plasmids.

Together with colistin, carbapenems are used as last-resort antibiotics. Ngbede et al. investigated carbapenem and colistin resistance in 583 non-duplicate Enterobacteriaceae isolates recovered from humans, animals, and the environment in Nigeria to find that 18.9% were resistant to at least one carbapenem and 9.1% exhibited concurrent carbapenem-colistin resistance. No carbapenem-resistant isolates carried any known carbapenemase-producing gene. Whole-genome sequencing supported that concurrent carbapenem-colistin resistance was mediated by novel or previously described alterations in chromosomal efflux regulatory genes, particularly *mgrB* (M1V), *ompC* (M1_V24del), *ompK37* (I70M, I128M), *ramR* (M1V), and *marR* (M1V). In addition, alterations/mutations were detected in the *etpA, arnT, ccrB, pmrB* in colistin-resistant bacteria and *ompK36* in carbapenem-resistant bacteria. In contrast, Azam et al. found that among 11 colistin-resistant *K. pneumoniae* isolated from humans in India, present non-synonymous potentially deleterious mutations in the *phoP, phoQ, pmrA, pmrB*, and *mgrB* genes, and amongst those, one clone carried the New Delhi metallo-beta-lactamase 1 gene (NDM-1) and expressed carbapenem resistance.

*Salmonella* is one of the leading causes of global bacterial food poisoning worldwide. However, the occurrence and transmission of *mcr* genes in *Salmonella* are still poorly understood. Hu et al. analyzed 755 foodborne *Salmonella* from 26 provinces in mainland China in 2016. As a result, 10% of the isolates were defined as multidrug-resistant (MDR), and two carried *mcr-1*: *S*. Derby CFSA231 and *S*. Typhimurium CFSA629. Both expressed an MDR phenotype and included a single circular chromosome and one plasmid. Among the 22 AMR genes identified in S. Derby CFSA231, only the *mcr-1* gene was localized on the IncX4 type plasmid pCFSA231 while 20 chromosomal AMR genes, including four plasmid-mediated quinolone resistance (PMQR) genes, were mapped within a 64 kb *Salmonella* genomic island (SGI) like region. *S*. Typhimurium CFSA629 possessed 11 resistance genes including an *mcr-1.19* variant and two ESBL genes. The contribution by Vázquez et al. shows that 2.2% of foodborne *Salmonella* isolated in Asturias, Spain, between 2014 and 2019 were resistant to colistin. Four of these isolates, belonging to the European monophasic ST34 clone of *S*. Typhimurium characterized by chromosomal genes conferring resistance to ampicillin, streptomycin, sulfonamides, tetracycline, heavy metals, and arsenic ± mercury, carried *mcr-1* in IncX4 or IncHI2 plasmids. Moreover, Diaconu et al. reported that 6.2% of AMR isolates from foodstuffs belonging to the same ST34 clone presented the *mcr-9* gene carried by IncHI2 megaplasmids of near 300 Kb, associated with increased resistance to colistin.

Previously unknown determinants for colistin resistance are starting to be revealed. In addition to chromosomal mutations affecting the two-component sensory systems (TCSS) PhoPQ or PmrAB, and the *mcr* genes, modification of lipopolysaccharide lipid A with aminoarabinose (L-Ara4N) is carried out by the enzymes encoded in *arn* operons (Figure 1). These modifications might confer colistin resistance upon up-regulation by chromosomal mutations of TCSS and can also be plasmid-mediated, similarly to *mcr* genes (Gallardo et al., 2021). Some Gram-negative bacteria are intrinsically resistant to very high levels of colistin, and the work by Panta and Doerrler evidenced that a DedA family protein in *Burkholderia thailandensis* (DbcA; DedA of B*urkholderia* required for colistin resistance) is a membrane transporter required for resistance to colistin, which function is partially restored by overexpression of *arn* genes or by the increase in membrane potential that can occur by lowering the pH.

**Figure 1:**
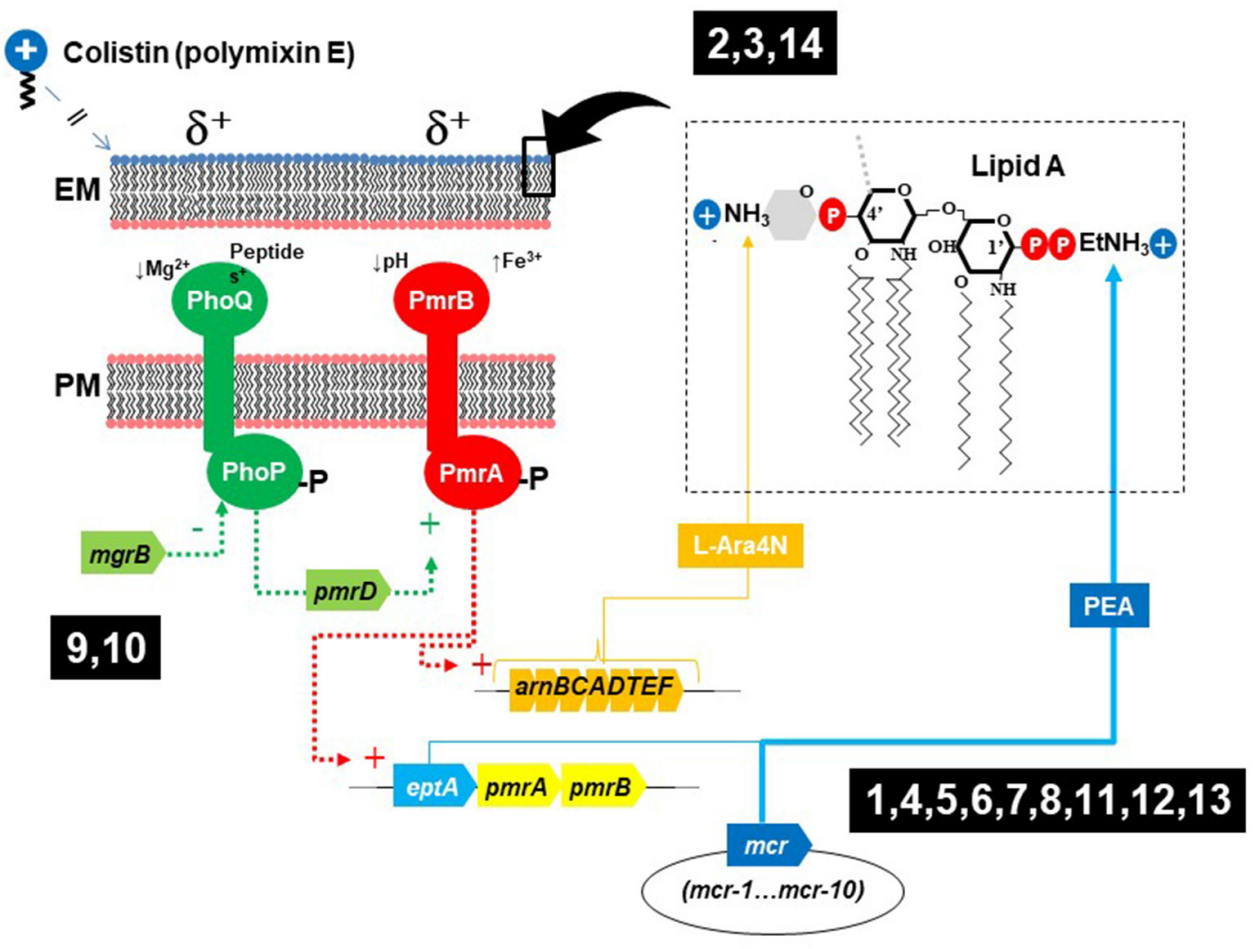
Elements involved in colistin resistance in enterobacteria, biological context of the articles published in this research topic. (1, Cheng et al.; 2, Zhu et al.; 3, Miguela-Villoldo et al.; 4, Hiba Al-Mir et al.; 5, Nakano et al.; 6, Savin et al.; 7, Lopes et al.; 8, Zhang et al.; 9, Ngbede et al.; 10, Azam et al.; 11, Hu et al.; 12, Vázquez et al.; 13, Diaconu et al.; 14, Panta and Doerrler).

Published contributions on this topic are indicated in Figure 1 in relation to their context on colistin resistance (1, Cheng et al.; 2, Zhu et al.; 3, Miguela-Villoldo et al.; 4, Hiba Al-Mir et al.; 5, Nakano et al.; 6, Savin et al.; 7, Lopes et al.; 8, Zhang et al.; 9, Ngbede et al.; 10, Azam et al.; 11, Hu et al.; 12, Vázquez et al.; 13, Diaconu et al.; 14, Panta and Doerrler).

## Author Contributions

All authors listed have made a substantial, direct, and intellectual contribution to the work and approved it for publication.

## Funding

Work in the laboratory of AQ is funded by the Spanish Ministry of Science and Innovation (AEI, Spain), the Junta de Extremadura, and FEDER (Grants PID2020-118405RB-I00, IB20181, and grupo CTS059). AM acknowledges funds from the Agencia Estatal de Investigación (AEI, Spain), the Xunta de Galicia, and FEDER (Grants PID2019-104439RB-C21/AEI/10.13039/501100011033 and ED431C 2021/11). Work in the laboratory of MC is funded by the Portuguese Foundation for Science and Technology (FCT) through the strategic project UID/04292/2020 granted to MARE—Marine and Environmental Sciences Centre.

## Conflict of Interest

The authors declare that the research was conducted in the absence of any commercial or financial relationships that could be construed as a potential conflict of interest.

## Publisher's Note

All claims expressed in this article are solely those of the authors and do not necessarily represent those of their affiliated organizations, or those of the publisher, the editors and the reviewers. Any product that may be evaluated in this article, or claim that may be made by its manufacturer, is not guaranteed or endorsed by the publisher.
